# The way from stakeholders’ needs research to the public health policy and practice

**DOI:** 10.1093/eurpub/ckac129.443

**Published:** 2022-10-25

**Authors:** K Lewtak, L Sugay

**Affiliations:** Department of Health Promotion and Chronic Diseases, National Institute of Public Health – National Institute of Hygiene, Warsaw, Poland

## Abstract

The implementation of the project “ProfiBaza” was the unique opportunity to effectuate a data set with the possibility to showcase for the first time in Poland which public health (PH) interventions are taken and whether they cover population health needs. In order to be forethoughtful to this challenge, the needs of stakeholders within the health system were investigated. Particularly, considering the widely recognised knowledge-practice gap in the PH field. We would like to demonstrate the results of a needs analysis related to: the accessibility assessment of the information about PH interventions; the need of establishing a good practices database regarding health promotion and disease prevention; the possibility to use research findings in PH practice. All those would be presented from the perspective of researchers, politicians, decision makers and PH institutions. This research was conducted as a questionnaire-based survey and focus group interviews. Researchers were asked to assess the compatibility between the PH research and the population health needs. The group of health sector decision makers were questioned about the usage of the information available in the ProfiBaza for the process of evidence-based decision making; local government politicians - to assess their readiness to implement health programmes, use the good practices database and build partnerships for health while representatives of various sectors - to assess the need of initiating a knowledge translation platform via the launched ProfiBaza system. The results of the research demonstrate that there is a need for: solidified collaboration between different types of PH stakeholders, mobilization, coordination, sharing experiences, creating networks of cooperating institutions, which will enable the effective response to emerging challenges and opportunities and to counteract the phenomenon of dispersion of efforts in improving health and reducing social inequalities in health.

